# The Effects of a Digital Well-Being Intervention on Patients With Chronic Conditions: Observational Study

**DOI:** 10.2196/16211

**Published:** 2020-01-10

**Authors:** Acacia C Parks, Allison L Williams, Gina M Kackloudis, Julia L Stafford, Eliane M Boucher, Ryan D Honomichl

**Affiliations:** 1 Happify New York, NY United States; 2 Case Western Reserve University Cleveland, OH United States

**Keywords:** chronic illness, happiness, subjective well-being, psychology, positive, internet-based intervention, mobile apps

## Abstract

**Background:**

Chronic conditions account for 75% of health care costs, and the impact of chronic illness is expected to grow over time. Although subjective well-being predicts better health outcomes, people with chronic conditions tend to report lower well-being. Improving well-being might mitigate costs associated with chronic illness; however, existing interventions can be difficult to access and draw from a single theoretical approach. Happify, a digital well-being intervention program drawing from multiple theoretical traditions to target well-being, has already been established as an efficacious means of improving well-being in both distressed and nondistressed users.

**Objective:**

This study aimed to compare change in well-being over time after using Happify for users with and without a chronic condition.

**Methods:**

Data were obtained from Happify users, a publicly available digital well-being program accessible via website or mobile phone app. Users work on tracks addressing a specific issue (eg, conquering negative thoughts) composed of games and activities based on positive psychology, cognitive behavioral therapy, and mindfulness principles. The sample included 821 users receiving at least 6 weeks’ exposure to Happify (ranging from 42 to 179 days) who met other inclusion criteria. As part of a baseline questionnaire, respondents reported demographic information (age and gender) and whether they had any of the prespecified chronic conditions: arthritis, diabetes, insomnia, multiple sclerosis, chronic pain, psoriasis, eczema, or some other condition (450 reported a chronic condition, whereas 371 did not). Subjective well-being was assessed with the Happify Scale, a 9-item measure of positive emotionality and life satisfaction. To evaluate changes in well-being over time, a mixed effects linear regression model was fit for subjective well-being, controlling for demographics and platform usage.

**Results:**

At baseline, users with a chronic condition had significantly lower subjective well-being (mean 38.34, SD 17.40) than users without a chronic condition (mean 43.65, SD 19.13). However, change trajectories for users with or without a chronic condition were not significantly different; both groups experienced equivalent improvements in well-being. We also found an effect for time from baseline (*b*=0.071; SE=0.010; *P*<.01) and number of activities completed (*b*=0.03; SE=0.009; *P*<.01), and a 2-way interaction between number of activities completed and time from baseline (*b*=0.0002; SE=0.00006; *P*<.01), such that completing more activities and doing so over increasingly longer periods produced improved well-being scores.

**Conclusions:**

Data from this study support the conclusion that users with a chronic condition experienced significant improvement over time. Despite reporting lower subjective well-being on the whole, their change trajectory while using Happify was equivalent to those without a chronic condition. Consistent with past research, users who completed more activities over a longer period showed the most improvement. In short, the presence of a chronic condition did not prevent users from showing improved well-being when using Happify.

## Introduction

### Background

According to the Center for Disease Control, chronic conditions are the leading cause of death and disability in the United States. Chronic conditions affect over 40% of the US population [1], and almost 20% of individuals with chronic illnesses report activity limitations that impede their ability to complete daily tasks, including work [2]. Indeed, having a self-reported chronic condition is associated with decreased work function [3] and appears to be the strongest predictor of absenteeism and work impairment [4].

People with chronic conditions also account for the greatest use of health care services [2,5], accounting for 75% or more of health care costs [1]. Relative to people without chronic conditions, health care spending is 3 times higher for individuals with 1 chronic condition [2] and increases with each additional chronic condition [5] and the presence of activity limitations [2]. As a result of an aging population and increased life expectancy [5], the impact of chronic illness is expected to further grow over time [6].

Chronic conditions are associated with lower levels of subjective well-being, which is defined as a combination of greater positive affect and life satisfaction, and lower negative affect [7]. Decreasing levels of psychological well-being are also associated with increased risk for onset of chronic conditions [8]. For example, people suffering from insomnia report lower levels of subjective well-being [9], and people with asthma, arthritis, diabetes, or heart disease are more likely to indicate that they are dissatisfied or very dissatisfied with their lives [10]. Adolescents with chronic conditions also report poorer emotional well-being than those without chronic conditions [11].

Conversely, among individuals with chronic conditions, high levels of well-being benefit their physical health. Positive affect, eg, may improve survival and recovery rates among people with physical illnesses by activating the autonomic nervous system and the hypothalamic-pituitary-adrenal axis [12]. Positive mood increases the use of self-management strategies among individuals with chronic illness (eg, medication adherence, lifestyle changes, and engaging in preventative behaviors), resulting in fewer complications, symptoms, and activity limitations associated with that illness and, in turn, further boosts positive mood [13]. Greater emotional well-being also predicts better long-term prognoses among patients with physical illnesses [12]; in cardiac patients specifically, an increase of 1 SD in psychological well-being is associated with an 11% reduction in rehospitalization risk [14]. Higher levels of positive affect have demonstrated benefits (eg, better health outcomes and slower disease progression) in a number of other chronic conditions including HIV, chronic heart disease, and coronary artery disease [14-16].

Given the benefits of subjective and psychological well-being among people with chronic conditions, which has been demonstrated in both correlational and experimental studies, a growing body of research has examined the impact of interventions targeting well-being, or mental health, on chronic illness symptoms. A total of 3 key theoretical traditions have been leveraged to improve well-being and mental health in chronic conditions: cognitive behavioral therapy (CBT) [17], mindfulness-based stress reduction (MBSR) [18], and positive psychological interventions (PPIs) [19-21]. In patients living with chronic pain, a robust literature has found that it is possible to enhance subjective well-being, and as a result, to improve pain levels and lessen disability by delivering CBT [22], MBSR [23], or PPIs [24]. Indeed, across a number of chronic somatic diseases, MBSR has a positive impact on mental health and physical health outcomes [25-27], as does CBT [28,29]. Positive psychology approaches differ from CBT and MBSR in that they explicitly target positive affect, which has been associated with lower mortality rates, better treatment compliance, and slower disease progression in a variety of diseases, above and beyond the impact of depression [12,14,30,31]. Interventions that target positive affect have directly improved chronic pain symptoms [24,32,33], even in individuals with more severe disability such as spinal cord injury, neuromuscular disease, or multiple sclerosis [34].

However, there are numerous barriers to accessing in-person interventions, including cost, logistics, and stigma [35,36]. As a result, research has pivoted to explore internet-based interventions, which have the ability to widely increase access to treatment. It is now well established that internet-based behavioral interventions can have a positive effect on psychological well-being [37-39]. However, 1 limitation of previous research is that previous studies targeting well-being tend to draw on either CBT [28,29], mindfulness [23,25,27], or positive psychology [24,32,33], with studies including multiple theoretical approaches comparing those interventions rather than combining them [26]. However, we argue that it is important to embrace all 3 approaches for 2 reasons. First, there is some research to suggest that what works for one patient may not work for all patients; person-activity fit matters. It seems particularly important that the patient believes in the intervention they are using; it must feel authentic to them, and they must also think its premises are plausible [40]. Although mindfulness may be a panacea for one person, to another, it may sound corny. Similarly, CBT may seem overly intellectual to some, whereas it may be just the analytical approach that another patient was looking for. When a patient can choose between multiple approaches, they have the opportunity to select one that they feel is a fit for them, improving their chances of success [41]. Second, we believe that it is important to offer a packaged approach containing multiple frameworks because it seems clear that different psychological interventions operate via different mechanisms (eg, positive affect vs depression) and that intervening through both mechanisms could be better than intervening via only one [42,43]. To our knowledge, no other study or intervention has combined these methods.

### Study Objectives

In this study, we offered a digital intervention platform, Happify, which contains activities that draw from each of the 3 key theoretical approaches. The activities on Happify are adapted efficacious interventions, ie, interventions with evidence from at least two separate research studies, in different samples [44]. Activities are categorized into 5 different groups: savor (activities focusing on mindfulness), thank (activities focusing on gratitude), aspire (activities focusing on optimism, goal setting, and finding meaning and purpose), give (activities focusing on kindness, forgiveness, and prosociality), and empathize (activities focusing on self-compassion and perspective taking). On Happify, activities from the various categories are grouped into tracks, which are designed to focus on a specific issue or problem (eg, reducing stress). Users are able to freely choose a track of interest on the platform and to select between different activity variants in a track. Thus, by completing activities on the Happify platform, users are exposed to well-being interventions from the theoretical traditions of mindfulness, CBT, and positive psychology.

Prior research has demonstrated that using Happify can effectively increase subjective and psychological well-being. Moreover, 1 study of existing Happify users demonstrated that usage was associated with more than a 27% increase in positive emotions over the course of 8 weeks, with greater gains among high-usage participants [45]. In another study using a randomized controlled design, participants randomly assigned to Happify and who completed a minimum of 2 activities per week on average showed statistically greater improvements in depression, anxiety, and resilience compared with a psychoeducation comparison condition or participants with lower platform usage [46]. In addition, a recent study [47] conducted with employees who were experiencing high levels of emotional or workplace distress found that those who used Happify at the recommended level showed greater improvements in resilience than those randomized to a psychoeducational comparison condition or those who did not use their assigned platform. The ideal *dosage* identified in previous internal and published research is 16 activities over the course of 8 weeks [46,47]. Taken together, these results suggest that using Happify can improve well-being in a variety of contexts.

In summary, we argue that improving subjective well-being is important for individuals with chronic conditions because it can help improve their physical condition, thereby reducing the associated costs [1]. We also argue that existing interventions that target well-being can be difficult to access, as they are often offered in person, with associated expenses and other barriers [35,36], and are rarely integrated to contain multiple existing, evidence-based intervention approaches. We provide evidence that a digital platform, Happify, which draws from multiple theoretical traditions to target well-being, has already been established as an efficacious means of improving well-being in both distressed and nondistressed users [45-47]. Although individuals with chronic conditions tend to have lower levels of well-being compared with individuals without chronic conditions [7], there is no reason to believe that they will show a less robust response to these interventions.

In this study, we tested the hypothesis that Happify’s efficacy on users without chronic conditions would generalize to a sample of users who report living with a chronic condition. Specifically, we analyzed observational data using Happify to compare the trajectory of change in well-being over time experienced by users on Happify who do and do not report having chronic conditions.

## Methods

### Recruitment and Sample

Data were drawn from registered users of Happify, a publicly available digital platform that offers games and activities based on research in positive psychology, CBT, and mindfulness. Although Happify is located in the United States, the platform is available worldwide and has been localized in 8 different languages to date. Of the 821 users included in our analyses, the majority used the English language version of the platform (605/821, 73.7% of sample); the remaining users used Happify as translated into German (25/821, 3.1% of sample), Spanish (15/821, 1.8% of sample), Japanese (8/821, 1.0% of sample), French (4/821, 0.5% of sample), Portuguese (3/821, 0.4% of sample), and Chinese (1/821, 0.1% of sample).

When registering with Happify, users provided semipassive consent that their data could be used for research purposes. Specifically, to access Happify content, users were asked to agree to the following statement: “Information that we collect about you also may be combined by us with other information available to us through third parties for research and measurement purposes, including measuring the effectiveness of content, advertising, or programs. This information from other sources may include age, gender, demographic, geographic, personal interests, product purchase activity or other information.” Data from all users aged 18 years and older who created accounts on the site between October 29, 2018, and April 4, 2019 (when data were queried), were initially considered; before October 29, 2018, Happify did not ask users about their chronic condition status. Our secondary analysis of Happify consumer data was performed under the supervision of IntegReview, an independent institutional review board.

### Materials and Procedures

Screenshots of Happify can be found in a previous publication [46]. After registering with Happify, users completed the onboarding process by responding to a series of questions about their inter- and intrapersonal circumstances, as well as demographic questions such as gender and age, which were collected as a categorical variable with the following options: 18 to 24 years, 25 to 34 years, 35 to 44 years, 45 to 54 years, 55 to 64 years, and 65 years or older. This was completed to allow for the algorithmic recommendation of a one of a number of *tracks* focused on certain psychosocial topics such as health and well-being, mindfulness and meditation, and relationships. In addition, respondents were asked to select all that apply from a list of chronic conditions, including arthritis, diabetes, insomnia, multiple sclerosis, chronic pain, psoriasis, eczema, or some other condition. Respondents who selected “some other condition” were not asked to clarify further what that condition was. Finally, the respondent was asked to report on anxiety symptoms by completing the generalized anxiety disorder 2-item (GAD-2) scale [48].

### Primary Outcome: Subjective Well-Being

The respondent’s subjective well-being was assessed with the Happify Scale, a 9-item measure that includes a positive emotionality component and a life satisfaction component, with higher scores indicating greater well-being [45]. The 4-item subscale measuring positive emotionality was based on the Positive and Negative Affect Schedule [49], a self-report measure that asks participants to indicate the extent to which they experience positive and negative emotions. For example, using a 5-point scale ranging from “Never” to “Very often (almost every day),” participants were asked to respond to the following question, “In the past month, how often have you felt joyous, exuberant, inspired, and/or awestruck?” The 5-item life satisfaction subscale was adapted from the Satisfaction with Life Scale [50] and used to assess satisfaction with different life domains (eg, work, leisure, and relationships). For example, using a 7-point scale ranging from “Very dissatisfied” to “Very satisfied,” participants were asked to respond to the following question, “How satisfied do you feel with the relationships in your life?” Scores on the subjective well-being composite range from 0 (low subjective well-being) to 100 (high subjective well-being). Scale validation using a general population sample from Amazon MTurk indicated that scores between 46 and 49 corresponded to the 25th percentile, scores between 61 and 63 corresponded to the 50th percentile, and scores between 75 and 77 corresponded to the 75th percentile of the Happify Scale. Internal validation data indicated that composite scale scores had acceptable reliability (alpha=.89) and were significantly and strongly associated with both subjective happiness [51] at *r*=0.78 and a measure of depressive symptoms (Center for Epidemiologic Studies Depression Scale [52]) at *r*=−0.75.

Participants were prompted to complete the Happify Scale on the day after completing the platform registration onboarding process and every 2 weeks thereafter. In each case, the assessment was optional, and users were able to exit out of the assessment without completing it if they wished. As a result, there was considerable variability in terms of how many assessments users completed and when those assessments were completed. For each individual, we calculated an average time between any 2 assessments (in days). The average of this average across the sample is 30.69 days (SD=21.10), ranging from 11.83 to 149.

### Statistical Analysis

Descriptive statistics were stratified by self-reported chronic condition status (yes vs no). Group differences in baseline variables were examined using chi-square tests for categorical characteristics and *t* tests or Mann Whitney *U* tests (in the case of non-normally distributed variables) for continuous variables.

To evaluate changes in well-being over time, a mixed effects linear regression model was fit for subjective well-being. The predictor variable of key interest was self-report of any of the 8 chronic conditions gathered at baseline. A binary variable was created to indicate having 1 or more chronic conditions vs none. The following covariates were included as control variables: gender, age category, number of activities completed on Happify, baseline anxiety [48], and time from baseline to each assessment (in days). All of these were treated as fixed effects.

Normally distributed person-specific random effects were included to account for varying numbers of follow-up assessments. To test whether changes in outcome measures differed between those reporting a chronic condition and those not reporting a chronic condition, all interaction terms between time from baseline, number of activities completed, and chronic condition status were included. Adherence to modeling assumptions was tested using residual plots (eg, Q-Q plots to examine if residuals followed a Gaussian distribution) and was met.

All computations were done in R, version 3.6.1 [53]. All linear mixed models were fitted using the R packages *lme4* [54] and *lmerTest* [55]. *P* values were calculated from Satterthwaite approximations for degrees of freedom [56]. All tests were 2-sided, and *P* values less than .05 were considered statistically significant.

**Figure 1:**
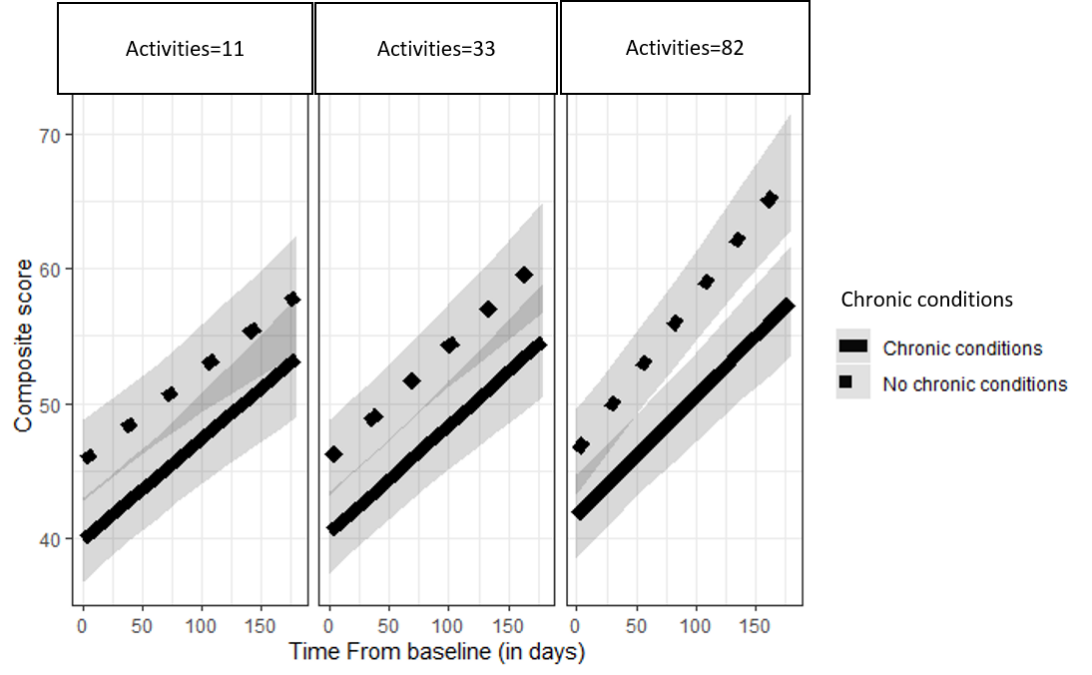
Change in well-being over time for users with and without a chronic condition. Facets are broken into the 25th, 50th, and 75th percentile of activities completed. Gray bands around the chronic condition lines reflect 95% CIs.

For Figure 1, data points are derived from predicted values of linear mixed models. Error bars are based on 95% CIs from those predicted values.

### Data Exclusion

During the study period, 6801 new users created accounts and completed the baseline assessment. Of these, data were excluded from those users who never completed any activities (n=1931) or who did not complete their self-report measures in a way that makes logical sense in relation to their usage of the platform (n=1058). Specifically, to be included, they were required to use Happify within 30 days of taking their initial baseline assessment—otherwise, their baseline assessment may or may not have accurately represented their state when they started using Happify. They were also required to have taken their final assessment within 30 days of their final activity to maximize chances that self-report scores were representative of the user’s psychological state when usage was terminated. In addition, users were excluded if they did not receive a minimum of 6 weeks’ exposure to Happify. They were not required to use Happify at any particular level during that time, but they were required to at least have had access to Happify for 6 weeks or more (2939 users were excluded by this criterion). Finally, 52 participants had missing onboarding questions because of a server error and were excluded. The final sample consisted of 821 users who had access to the platform between 42 and 179 days.

### Power

A statistical power analysis was performed for sample size estimation based on data from a randomized study examining Happify’s efficacy [46]. The effect size (ES) in this study, based on participants randomly assigned to Happify (vs a control condition) and who used the platforms at the recommended level (ie, completing an average of 2 activities per week), was *η*^2^=0.021, classified as small by Cohen [57] criteria. Specifying alpha=.05 and power=0.80, the projected sample size needed with this ES (GPower 3.1.9.2 [58]) for a repeated-measures analysis with a between-within interaction was n=94. This calculation was made with the conservative estimate of only 2 assessments and a correlation between repeated measures of *r*=0.50. Thus, this study was adequately powered to detect the effect of interest.

## Results

### Baseline Sample Characteristics

Table 1 displays the baseline sample characteristics of individuals who reported having a chronic condition and those who did not. Most importantly, the 2 groups were not statistically different with respect to the number of activities completed or the number of assessments finished. There were significant differences, however, between the 2 groups in terms of age, such that users reporting a chronic condition were more likely to fall into the older age categories and less likely to fall in the 18 to 24-year-old category. All users with a chronic condition reported having a health condition impacting their well-being, although 25 individuals without a chronic condition also reported this. Users with chronic conditions were also significantly more likely to be characterized as having anxiety based on their GAD-2 scores (65.6% vs 55.0%).

**Table 1:** Baseline sample characteristics and platform usage of the users with and without chronic conditions.

Characteristic		Chronic conditions (n=450)	No chronic conditions (n=371)	*P* value
Female, n (%)		372 (82.7)	305 (82.2)	.94
**Age (years), n (%)**				<.001
	18-24	73 (16.2)	103 (27.8)	
	25-34	139 (30.9)	116 (31.3)	
	35-44	109 (24.2)	88 (23.7)	
	45-54	86 (19.1)	41 (11.1)	
	55-64	35 (7.8)	20 (5.4)	
	65+	8 (1.8)	3 (0.8)	
Activities completed, mean (SD)		56.20 (86.63)	50.50 (64.51)	.29
Total time elapsed between first and last activity (in days), mean (SD)		72.19 (27.19)	76.23 (30.79)	.046
Total number of assessments, mean (SD)		4.18 (1.76)	4.20 (1.69)	.86
**Is there a health condition or concern that impacts your happiness or well-being currently?, n (%)**				<.001
	Not at all	0 (0.0)	346 (93.3)	
	Yes, not major	259 (57.6)	23 (6.2)	
	Yes, very much	191 (42.4)	2 (0.5)	
Arthritis, n (%)		31 (8.5)	0 (0.0)	<.001
Chronic pain, n (%)		75 (20.5)	0 (0.0)	<.001
Insomnia, n (%)		92 (25.2)	0 (0.0)	<.001
Multiple sclerosis, n (%)		3 (0.8)	0 (0.0)	.33
Psoriasis, n (%)		7 (1.9)	0 (0.0)	.05
Diabetes, n (%)		13 (3.6)	0 (0.0)	.003
Eczema, n (%)		0 (0.0)	0 (0.0)	Not applicable
Other chronic condition, n (%)		269 (73.7)	0 (0.0)	<.001
Number of chronic conditions, median (IQR)		1.00 (1.00-2.00)	0.00 (0.00-0.00)	<.001^a^
Positive emotion score, mean (SD)		32.34 (17.65)	36.43 (20.52)	.002
Life satisfaction score, mean (SD)		44.78 (21.55)	51.36 (22.55)	<.001
Subjective well-being, mean (SD)		38.34 (17.40)	43.65 (19.13)	<.001
Generalized anxiety disorder 2-item scores, median (IQR)		4.00 (2.00-6.00)	3.00 (2.00-5.00)	.002

^a^Mann Whitney *U* test.

The 2 groups were significantly different at baseline in terms of positive emotionality, as users with a chronic condition (mean 32.34, SD 17.65) had lower positive emotion scores than users without a chronic condition (mean 36.43, SD 0.52); life satisfaction baseline scores were similarly statistically different for users with a chronic condition (mean 44.78, SD 21.55) and those without a chronic condition (mean 51.36, SD 22.55), with users with a chronic condition scoring lower. Users with a chronic condition also had lower scores on the composite subjective well-being scale (mean 38.34, SD 17.40) than users without a chronic condition (mean 43.65, SD 19.13).

For those users with a chronic condition, the most common reported category was “other,” followed by insomnia, chronic pain, and arthritis. The most common number of reported conditions was 1; however, 136 users (136/450, 30.2% of the users with chronic conditions) reported having 2 or more.

### Change in Well-Being Over Time

For subjective well-being scores at final assessment, there were main effects for chronic condition status (*b*=4.82; SE=1.51; *P*<.01) and baseline GAD-2 score (*b*=−3.43; SE=0.30; *P*<.01). Users reporting a chronic condition and users reporting higher levels of anxiety had lower subjective well-being scores at their final assessment. In addition, there was an effect for time from baseline (*b*=0.071; SE=0.010; *P*<.01) and number of activities completed (*b*=0.03; SE=0.009; *P*<.01). Higher subjective well-being scores occurred among users who had been active users on Happify for longer and who had completed higher numbers of activities. Finally, there was a 2-way interaction between number of activities completed and time from baseline (*b*=0.0002; SE=0.00006; *P*<.01). For all users, completing more activities and doing so over increasingly longer periods interacted to produce improved well-being scores. There were no other significant interactions. However, there was no significant interaction between time from baseline and chronic condition status (*b*=−0.013; SE=0.071; *P*=.46). These results indicate that users with and without a chronic condition both experienced equal well-being improvements from using Happify and from completing higher numbers of activities on the platform; both groups of users showed the same pattern of change in well-being over time. Depictions of changes in the subjective well-being scale across time for both users with and without a chronic condition are presented in Figure 1. Level of activities completed is split into 3 facets for the 25th (activities completed=11), 50th (activities completed=33), and 75th (activities completed=82) percentiles.

## Discussion

### Principal Findings

A key objective of this study was to explore whether a digital intervention could reliably improve subjective well-being among users living with a chronic condition. We were particularly interested in testing the impact of an intervention that targets subjective well-being because of the demonstrated benefits of subjective well-being, and especially positive affect, among individuals with chronic conditions such as greater self-management of their condition [13], better long-term prognoses [24], and better health outcomes [7,12,14-16]. Although other research suggests that CBT, MBSR, and PPIs increase subjective well-being and improve physical health outcomes among people with chronic conditions [22-24], many of these interventions are in person, making scaling difficult. In addition, these other interventions draw on just a single theoretical approach [26], rather than combining strengths from all 3 approaches into a single intervention. We explored the impact of improving well-being on users with chronic conditions using observational data from Happify, an existing commercial platform that integrates principles from CBT, mindfulness, and positive psychology and contains users both with and without chronic conditions. Although prior research supports the idea that Happify improves well-being among physically healthy users [35,45-47], no research to date has tested Happify’s efficacy in users who are dealing with a chronic disease.

Consistent with other studies demonstrating the effectiveness of PPIs [32-34], mindfulness [23,26], and CBT [26] on subjective well-being among people with chronic conditions, data from this study support the conclusion that users with a chronic condition experienced significant improvement in subjective well-being over time, and their change trajectory did not differ from those without a chronic condition. Users who completed more activities over a longer period showed the greatest amount of improvement, a finding that is consistent with past research [45-47], and chronic condition status did not change this result. In other words, the presence of a chronic stressor, at least in the case of chronic conditions, does not appear to prevent users from experiencing improvements in well-being when they use Happify.

Although previous research typically focused on specific conditions such as chronic pain [32], spinal cord injury, multiple sclerosis, neuromuscular disease [34], or osteoarthritis [33], we used a noncategorical approach [59], grouping all users who self-reported a chronic condition together. Consequently, data from this study do not speak to whether effectiveness differed by type of condition. However, other studies found no differences in subjective well-being based on type of condition or visibility of the condition [11]. In addition, researchers have advocated for the use of a noncategorical approach in applied research on chronic conditions because people with chronic conditions share common problems that go above and beyond the specific symptoms of their particular illness [59], including lower subjective well-being. This approach may be particularly relevant when evaluating interventions as communities may have a large proportion of individuals with chronic conditions but only small numbers of people with specific conditions [59]. Arguably, then, by using a noncategorical approach and including participants with a variety of chronic conditions, our findings have more direct applicability to those suffering from these conditions.

### Future Directions

This study provides preliminary evidence that Happify can significantly improve subjective well-being among people with chronic conditions, despite the fact that people with chronic conditions also are more likely to suffer from more serious psychological distress. For example, although depression has a prevalence of 10% to 20% in the general population, among individuals living with a chronic condition, depression rates range from 35% to 50% [60-63]. Individuals with chronic pain are 4 times more likely to have either a depressive or anxiety disorder than those without chronic pain [64], and the incidence of comorbid depression and anxiety disorders is greater than independent diagnoses of either depression or anxiety [65]. People with insomnia are 2 times more likely to develop depression than those without insomniac symptoms [66], and chronic insomnia is associated with an elevated risk for anxiety disorders [67].

Importantly, the burden of chronic illness can be amplified when poor mental health, especially depression, is also present. Chronic pain patients with comorbid depression and anxiety report greater pain severity and pain-related disability as well as poorer health-related quality of life than people with pain alone [68]. Depression also predicts poor treatment adherence, greater frequency of complications, and higher mortality rates among people with diabetes [69,70]. In fact, in a study of 60 countries, respondents with a chronic physical condition and comorbid depression had the worst health scores overall [71]. Although previous research has shown that Happify users report fewer depressive and anxiety symptoms after 8 weeks [46], we only assessed subjective well-being in this study. Therefore, in future research, it will be important to determine whether Happify also helps to reduce depressive and anxiety symptoms among people with chronic conditions.

Another important direction for future research is to explore how, specifically, Happify usage helps to improve mental well-being. Previous research suggests that mindfulness programs have been effective in reducing depressive symptoms among individuals with chronic pain by reducing pain catastrophizing and psychological distress [72]. Other studies show that Web-based interventions lead to fewer activity limitations [73] and improved pain acceptance [23]. Future research should include measures assessing participants’ physical condition as well, including activity limitations, pain severity and acceptance, and pain catastrophizing, to determine whether Happify usage also impacts these outcomes and whether they mediate the relationship between Happify use and improved subjective well-being. Similarly, research exploring the long-term benefits of Happify on users’ psychological and physical well-being would be valuable to determine whether it, like some other interventions [74], can also help to lower costs associated with chronic conditions by reducing health care usage.

### Limitations and Strengths

This study was a naturalistic, observational study of existing Happify users. Although observational studies are an important tool in the assessment of health-related outcomes [75], there are also several limitations associated with the lack of control in these designs. One limitation is that although user data were collected in a realistic context, participants were all Happify registrants who made their way to the platform naturally and, consequently, may differ from people in the general population that do not use Happify. Specifically, this study and several others reliably find that users on Happify are more distressed than the general population. However, the sample is likely to be biased in the same direction as future Happify participants, so any conclusions drawn about this sample may well be applicable to our population of interest—ie, those who use Happify in the future.

Usage patterns observed in this study were also naturally occurring, as compared with those that may be observed in a more controlled study with participation incentives and more frequent (potentially annoying or invasive) reminders. Nevertheless, given that the Happify platform tested in this study is a commercial product, freely available to the public, and just as easy to quit as it is to sign up, dropout levels were higher than would be observed in a more controlled setting. The resulting sample consisted of only the most dedicated users. Therefore, self-selection is a concern for this type of study design. However, even randomized controlled trials (RCTs) can suffer from this, as unmeasured moderating variables may influence a participant’s willingness to participate in a randomized study [57].

Finally, because this study is observational, we cannot rule out the possibility that the users with a chronic condition were different from those without a chronic condition in ways we did not measure; as chronic condition status cannot be randomized, we did not have the benefit of randomization to address systematic biases. Moreover, although we collected respondent data on a number of chronic conditions, our list of chronic conditions was not exhaustive. Approximately 73.7% (605/821) of respondents in the chronic condition group self-identified as having “some other condition” for which we have no additional information. It may also be that users with and without a chronic condition differ on meaningful but unmeasured covariates. For example, as Happify is a commercial product and the analyses included in this study are secondary analyses from Happify’s consumer base and not a randomized clinical trial, we did not have access to user information that might be relevant here, such as access to other mental health-related treatments. Such differences between groups or omitted variables can contribute to biased estimates of treatment effects [76]. In addition, users in this study completed activities and assessments at varying times and to a varying degree. This also creates difficulty in assessing change over time compared with an RCT, where the intervention and assessments are planned and given at regular intervals. However, linear mixed models have been shown to be effective at controlling for such unbalanced data occurring at varying time points [77].

### Conclusions

It is all too easy in the world of digital well-being interventions, the use of which is largely unregulated, to assume that an intervention that works in one population can safely be generalized to other populations. We would argue that it is important to understand who may be in the sample of consumers interacting with a digital intervention and to evaluate whether there are subgroups of users for whom the intervention fails to produce results. Although in the case of this paper, we were able to ascertain that Happify’s effects on well-being do not differ significantly between users with chronic conditions and those without chronic conditions, we could also have found that users with chronic conditions need something else; only by evaluating subgroup data can we gain confidence in our ability to generalize, as a freely available digital intervention inevitably will. In summary, this study provides valuable observational evidence of the efficacy of Happify’s use among real users living with chronic conditions under naturalistic conditions. Given these data, future research should seek to replicate these effects under more controlled conditions, such as RCTs, and explore the impact of Happify’s use on other important outcomes associated with chronic conditions such as depressive and anxiety symptoms as well as physical and health-related outcomes.

## Abbreviations

CBT: cognitive behavioral therapy
ES: effect size
GAD-2: generalized anxiety disorder 2-item
MBSR: mindfulness-based stress reduction
PPIs: positive psychological interventions
RCT: randomized controlled trial
